# Impact of unlinked deaths and coding changes on mortality trends in the Swiss National Cohort

**DOI:** 10.1186/1472-6947-13-1

**Published:** 2013-01-04

**Authors:** Kurt Schmidlin, Kerri M Clough-Gorr, Adrian Spoerri, Matthias Egger, Marcel Zwahlen

**Affiliations:** 1 Division of International & Environmental Health, Institute of Social and Preventive Medicine (ISPM), University of Bern, Bern, Switzerland; 2 National Institute for Cancer Epidemiology and Registration (NICER), Institute of Social and Preventive Medicine (ISPM), University of Zürich, Zürich, Switzerland; 3 Section of Geriatrics, Boston University Medical Center, Boston, MA, USA; 4 Department of Social and Community Medicine, University of Bristol, Bristol, UK; 5 Institute of Social and Preventive Medicine (ISPM), University of Bern, Finkenhubelweg 11, Bern, CH-3012, Switzerland

**Keywords:** Cohort studies, Record linkage, Mortality, Trends

## Abstract

**Background:**

Results of epidemiological studies linking census with mortality records may be affected by unlinked deaths and changes in cause of death classification. We examined these issues in the Swiss National Cohort (SNC).

**Methods:**

The SNC is a longitudinal study of the entire Swiss population, based on the 1990 (6.8 million persons) and 2000 (7.3 million persons) censuses. Among 1,053,393 deaths recorded 1991–2007 5.4% could not be linked using stringent probabilistic linkage. We included the unlinked deaths using pragmatic linkages and compared mortality rates for selected causes with official mortality rates. We also examined the impact of the 1995 change in cause of death coding from version 8 (with some additional rules) to version 10 of the International Classification of Diseases (ICD), using Poisson regression models with restricted cubic splines. Finally, we compared results from Cox models including and excluding unlinked deaths of the association of education, marital status, and nationality with selected causes of death.

**Results:**

SNC mortality rates underestimated all cause mortality by 9.6% (range 2.4% - 17.9%) in the 85+ population. Underestimation was less pronounced in years nearer the censuses and in the 75–84 age group. After including 99.7% of unlinked deaths, annual all cause SNC mortality rates were reflecting official rates (relative difference between −1.4% and +1.8%). In the 85+ population the rates for prostate and breast cancer dropped, by 16% and 21% respectively, between 1994 and 1995 coincident with the change in cause of death coding policy. For suicide in males almost no change was observed. Hazard ratios were only negligibly affected by including the unlinked deaths. A sudden decrease in breast (21% less, 95% confidence interval: 12% - 28%) and prostate (16% less, 95% confidence interval: 7% - 23%) cancer mortality rates in the 85+ population coincided with the 1995 change in cause of death coding policy.

**Conclusions:**

Unlinked deaths bias analyses of absolute mortality rates downwards but have little effect on relative mortality. To describe time trends of cause-specific mortality in the SNC, accounting for the unlinked deaths and for the possible effect of change in death certificate coding was necessary.

## Background

Mortality, an important outcome in epidemiological studies, generally has to be ascertained over long follow-up periods. This can be achieved either via prospective active follow-up, which is labor intensive, expensive and potentially biased due to losses to follow-up, or via linkage to a regional or national death registry, which has become more frequent due to the electronic availability of registry data [1-6]. Incomplete enumeration of persons in a census, undocumented migration and data errors can, however, lead to incomplete linkage and incomplete mortality follow-up; which in turn might introduce bias in analyses of all cause and cause-specific mortality rates and determinants of mortality [7,8]. Incomplete mortality ascertainment leads to an underestimation of mortality rates mainly because the total number of deaths is too small (not all deaths are counted) and because the total person-time is too large (person-time under observation is not stopped without a date of death).

When the focus is on cause-specific mortality rates (e.g. site-specific cancer mortality) additional issues relating to the cause of death classification need to be considered. Changes in cause of death coding policy, for example switching from one version of the International Classification of Diseases (ICD) to another, can affect the time trends of cause-specific mortality rates, as previously documented for respiratory diseases, circulatory diseases and cancer [9-14]. In older age-groups, where mortality is highest, both unascertained deaths and coding changes may dramatically affect absolute rates.

We investigated the bias introduced by incomplete ascertainment of deaths and changes in coding in the Swiss National Cohort (SNC) [15,16], a census based cohort study where mortality ascertainment is performed via linkage to the national death registry with about 95% completeness. We included the unlinked deaths using a pragmatic linkage algorithm and used Poisson regression models to account for changes in Swiss Federal Statistical Office (SFSO) coding of causes of death.

## Methods

### Swiss National Cohort (SNC)

The anatomy of the SNC has been described in detail elsewhere [15]. Briefly, the SNC is a longitudinal study of the entire resident population of Switzerland, based on national census information. The SNC includes 6.8 million people at the census 1990 and 7.3 million at the census 2000. Regularly updated mortality and migration files are linked with the census 1990 and 2000. In the period 1991–2000 621,389 death certificates were recorded by the national death registry at the SFSO and 432,004 certificates were recorded in the period 2001–2007 for a total of 1,053,393 deaths. In the absence of a unique personal identifier, both deterministic and probabilistic methods of record linkage based on sex, date of birth, marital status, religion, nationality, place of residence and other variables when available (e.g. date of birth of mother or spouse) were used. If the census and death record that refer to the same person are recorded several years apart, then place of residence, marital status and nationality could have changed and will disagree on the two records. Linkage will be less successful, depending on the level of changes in these characteristics. Causes of death were coded at the national death registry of the SFSO according to the eighth revision of the ICD (ICD-8) until 1994 and according to the 10th revision (ICD-10) since 1995. Ethical approval was obtained from the Ethics Committees of the Cantons of Zurich and Bern.

### Unlinked deaths

Among the 1,053,393 deaths recorded between 5^th^ December 1990 and 31^st^ December 2007 56,413 (5.4%) could not be linked to a census or migration record. Deaths relating to persons born between censuses were not considered as unlinked (e.g. a 1998 death of a child born in 1994 was not linkable to the SNC population because the child was born after census 1990 and died before census 2000). Deaths that could not be linked were younger at death, less likely to be Swiss nationals and more likely to be women and single, as described in detail elsewhere [15].

We implemented a pragmatic two-step procedure to allocate unlinked death records to census records. We applied rules to prevent impossible matches, for example when attributing deaths with a gender specific cause of death (e.g. prostate or breast cancer). In a first step we used the following procedure to allocate unlinked deaths to census records: death and census record matched on gender, canton of residence, nationality, age (same birth date or maximally 3 months apart), civil status (identical or plausible change, such as married at census and widowed at time of death). If more than one census record fulfilled these criteria, we randomly allocated the death to one of them. If no census record was found, we used less stringent matching criteria in a second step: gender, region (Central, Eastern, Zurich, the Espace Mittelland, Lake Geneva, Northwestern, or Ticino) and birth date within one year. We again randomly selected one census record that matched the death record on these criteria.

### Official mortality rates and SNC rates including and excluding unlinked deaths

We first calculated age- and gender-specific official cause-specific mortality rates by dividing all deaths of a specific cause of death recorded in Switzerland by the official midyear population data from the SFSO for each year of the period 1991–2007 (for males and females and 10 year age categories up to age 84 and a final category of the 85+ age group). These rates are hereafter referred to as reference rates.

We then calculated age- and gender-specific mortality rates based on the SNC (hereafter SNC rates), measuring time from the date of the census (5^th^ December 1990 or 5^th^ December 2000) to either the date of death, date of emigration, or 31^st^ December 2007, whichever came first. We calculated the total person-time separately for each calendar year 1991–2007, gender and age-group and divided the corresponding number of deaths by the number of person-years. We did calculations both including and excluding the unlinked deaths.

We show results for selected causes of death: deaths from all causes and for all cancer causes (ICD-8: 140–209, ICD-10: C00-C97), all cardiovascular causes (ICD-8: 390–429, ICD-10: I00-I52), and suicides (ICD-8: E950-E959, ICD-10: X60-X84). As over 50% of deaths occur in the age-groups 75–84 years and 85+ years, we provide descriptive statistics for the percentage difference between the two versions of SNC rates (excluding or including unlinked deaths) and the reference rate for age-groups 75–84 years and 85+ years.

### Accounting for change in official cause of death coding policy

In Switzerland and elsewhere the underlying cause of death on the death certificate is defined as “(a) the disease or injury which initiated the train of morbid events leading directly to death, or (b) the circumstances of the accident or violence which produced the fatal injury” [17] and is generally considered the most meaningful cause from a public health standpoint. Although the notion of the underlying cause of death appears to be straight-forward, the determination of the sequence of causes may be difficult when a number of diseases and conditions are involved. The reporting physicians can list up to four additional diseases related to the death of the person. This information is used by the SFSO to assign the official cause of death. Through 1994 the SFSO official cause of death coding policy used ICD-8 combined with internal rules giving priority to some causes (accident, poisoning or trauma; influenza; cancer). In 1995, SFSO changed to ICD-10 and decided to strictly follow ICD coding [14]. A sudden change in mortality rates between 1994 and 1995 was observed, most pronounced in cancers with long survival (e.g. breast and prostate cancer) [14,18]. For example, from 1995 onwards the mention of breast cancer on the death certificate of an elderly woman resulted less often in breast cancer being the official cause of death than in the preceding years [18].

We used Poisson regression models that included a variable to account for the change in rates resulting from the 1995 change in coding of causes of death. We modeled the natural logarithm of the number of events and included the natural logarithm of the person-time at risk as a fixed offset [19]. The dataset consisted of records for each calendar year between 1991–2007 with the number of deaths (all cause or cause-specific) and the person-time at risk calculated from the SNC for males and females for a specific age category. We included restricted cubic splines using predefined equally spaced connecting knots at 1990, 1995, 2000, 2004 to flexibly model time trends of absolute rates [20,21]. These models allowed estimating absolute mortality rates with 95% confidence intervals (95% CI) for the years before 1995 as if the post-1995 official cause of death coding policy had been used during the earlier years. In addition the estimated parameter for the sudden change in official cause of death coding policy can be understood as a multiplication factor with which the rate calculated in the year 1994 would need to be multiplied to be comparable to rate calculated in the year 1995. We illustrate the impact of the change in coding policy for breast cancer (ICD8: 174–175, ICD10: C50), prostate cancer (ICD8: 185, ICD10: C61), all cancer causes (ICD8: 140–209, ICD10: C00-C97), and suicides (ICD: E950-E959, ICD10: X60-X84) for age-groups 75–84 years and 85+ years. We also present the estimated multiplication factors and their 95% CI.

### Hazard ratios by education, marital status and nationality

We analyzed the association of education, marital status, and nationality with all cause, all cancer, all cardiovascular, and suicide mortality using multivariable Cox regression models. We investigated how estimated hazard ratios (HR) differed if we included or excluded unlinked deaths in the analysis. In addition to education, marital status and nationality all models included the categorical variables language region, religion, and degree of urbanization of the place of residence. All analyses were done using Stata 11.1 and 12.1 (StataCorp, College Station, Texas).

## Results

### Unlinked deaths

Table 1 shows the socio-demographic characteristics of all deaths and unlinked deaths 2001–2007 and of the census 2000 population. Children and younger adults (age <34 years) had a higher proportion of unlinked death records. In absolute numbers, however, more unlinked deaths occurred in older age-groups. The percentage of unlinked deaths was slightly higher in women and the percentage was markedly higher in foreigners (12.2% compared to 4.7% in Swiss). Within categories of marital status, the percentage of unlinked deaths was highest in singles.

**Table 1:** Characteristics of all deaths and unlinked deaths between 2001 and 2007 and of the population from census 2000

	**Deaths**		**Census population**
	**All(N,column%)**	**Unlinked(N,row%)**	**(N,column%)**
**Total**	487,730 (100%)	25,587 (5.2%)	7,279,556 (100%)
**Gender**			
Women	252,232 (51.7%)	12,276 (4.8%)	3,715,863 (51.0%)
Men	235,498 (48.3%)	13,311 (5.7%)	3,563,693 (49.0%)
**Age at census 2000(years)**			
0-14	1,842 (0.4%)	300 (16.3%)	1,249,271 (17.2%)
15-24	3,552 (0.7%)	926 (26.1%)	851,735 (11.7%)
25-34	5,847 (1.2%)	1,300 (22.2%)	1,080,403 (14.8%)
35-44	13,437 (2.8%)	1,765 (13.1%)	1,192,374 (16.4%)
45-54	28,387 (5.8%)	2,479 (8.7%)	999,661 (13.7%)
55-64	52,063 (10.7%)	2,996 (5.8%)	792,783 (10.9%)
65-74	99,195 (20.3%)	3,870 (3.9%)	587,723 (8.1%)
75-84	167,438 (34.3%)	7,093 (4.2%)	381,520 (5.2%)
85-94	108,044 (22.2%)	4,606 (4.3%)	135,542 (1.9%)
95+	7,925 (1.6%)	252 (3.2%)	8,544 (0.1%)
**Nationality**			
Swiss	451,691 (92.6%)	21,174 (4.7%)	5,786,075 (79.4%)
Non-Swiss	36,039 (7.4%)	4,413 (12.2%)	1,493,481 (20.5%)
**Educational attainment**			
Compulsory schooling	251,032 (51.5%)	11,850 (4.7%)	2,206,231 (30.3%)
Secondary education	184,883 (37.9%)	9,852 (5.3%)	2,802,202 (38.5%)
Tertiary education	49,989 (10.2%)	3,587 (7.2%)	1,027,008 (14.1%)
Not applicable	1,826 (0.4%)	298 (16.3%)	1,244,115 (17.1%)
**Marital status**			
Single	65,296 (13.4%)	5,097 (7.8%)	3,061,239 (42.0%)
Married	224,196 (46.0%)	11,763 (5.2%)	3,396,553 (46.7%)
Widowed	162,726 (33.4%)	6,753 (4.1%)	414,316 (5.7%)
Divorced	35,512 (7.3%)	1,974 (5.6%)	407,448 (5.6%)
**Type of household**			
Single person	156,395 (32.1%)	8,905 (5.7%)	1,120,857 (15.4%)
Multi persons	258,349 (53.0%)	13,720 (5.3%)	5,864,661 (80.6%)
Institution	72,986 (15.0%)	2,962 (4.1%)	294,038 (4.0%)
**Language region**			
German	352,101 (72.2%)	15,740 (4.5%)	5,241,017 (72.0%)
French	112,273 (23.0%)	8,214 (7.3%)	1,718,363 (23.6%)
Italian	23,356 (4.8%)	1,633 (7.0%)	320,176 (4.4%)
**Religion**			
Protestant	226,674 (46.5%)	9,616 (4.2%)	2,567,228 (35.3%)
Roman Catholic	192,487 (39.5%)	10,307 (5.4%)	3,045,563 (41.8%)
No denomination	31,335 (6.4%)	2,522 (8.0%)	809,202 (11.1%)
Other / unknown	37,234 (7.6%)	3,142 (8.4%)	857,563 (11.8%)
**Urbanization(Community of residence)**			
Urban	164,540 (33.7%)	8,550 (5.2%)	2,075,713 (28.5%)
Periurban	192,438 (39.5%)	10,700 (5.6%)	3,263,588 (44.8%)
Rural	130,752 (26.8%)	6,337 (4.8%)	1,940,255 (26.7%)

We allocated almost all (56,265; 99.74%) unlinked death records from the years 1991 to 2007 to a census record. Only 148 could not be linked; 144 related to census 1990 (deaths in 1991–2000) and four to census 2000 (deaths in 2001–2007). This represents 0.26% of all unlinked deaths (148 of 56,413), and 0.014% of all deaths (148 of 1,053,393). All 148 unassigned death records were in the elderly (>75 years).

The agreement between information on census and on death certificate was high for the main SNC linkage: 99.95% for sex, 97.1% for the exact date of birth, 99.1% for nationality, 92.0% for marital status and 89.4% for community of residence (Table 2). With the exception of sex, agreement was much lower for the additional pragmatic linkages of initially unlinked deaths. For example, the date of birth matched in about 50% in step 1 and in less than 1% in step 2.

**Table 2:** Agreement between information on census record and on death certificate for key variables for the probabilistic main SNC linkage and for the additional pragmatic linkages of deaths

**Variable**	**Mortality and census records with identical value of variable (%)**		
	**SNC linkage**^Ϯ^	**Linkage of unlinked deaths(step1)**	**Linkage of unlinked deaths(step 2)**
	(n=996,980)	(n=51,002)	(n=5,263)
**Sex**			
(% identical)	99.95%	100.0%	100.0%
**Date of birth**			
(% identical)	97.1%	53.2%	0.3%
(Difference in days: 25^th^ percentile, median, 75^th^ percentile)	(0, 0, 0)	(0, 0, 0)	(−109, -12, 96)
**Marital status**			
(% identical)	92.0%	79.3%	55.7%
(% identical or plausible change*)	99.7%	100.0%	87.9%
**Nationality**			
(% identical)	99.1%	100.0%	95.9%
**Place of residence**			
(% identical)	89.4%	23.0%	11.3%

^Ϯ^ Done by probabilistic linkage which attributes different log(likelihood ratios) for being the same person based on full agreement, full disagreement, plausible change, or possible recording error of information on sex, date of birth, marital status, nationality, place of residence, religion, date of birth of wife/husband (if available) etc.

* Plausible changes are single to married, married to divorced, married to widowed, divorced to married, widowed to married.

### Comparison of absolute mortality trends

**Figure 1:**
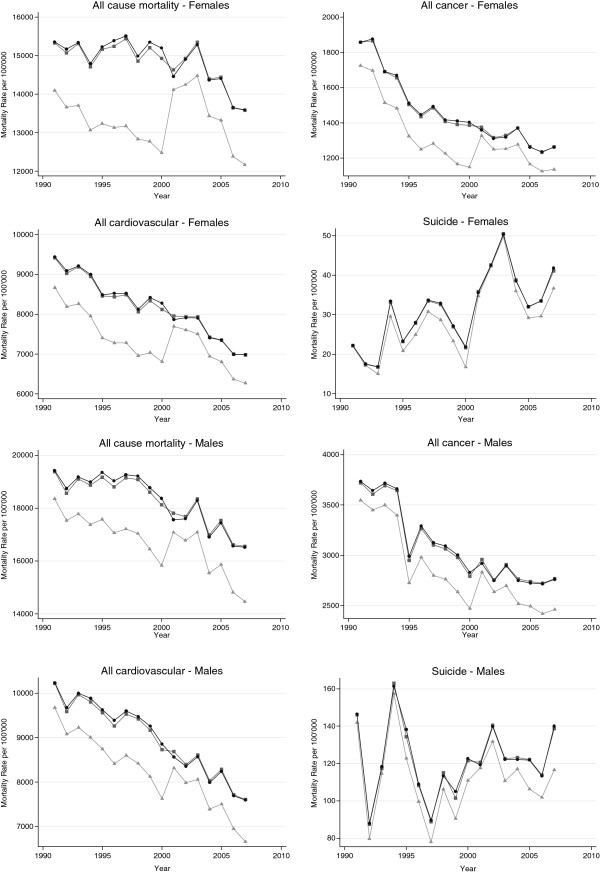
Uncorrected and corrected SNC mortality rates for selected causes of death in comparison to the Swiss reference rates for the population aged 85 and older*. Light gray triangles: uncorrected rate SNC, dark gray squares: rate SNC after allocation, black dots: reference rate. *Uncorrected SNC rates were calculated with SNC death certificates linked to census 1990 and 2000 (numerator) and exact person-time at risk (denominator). The corrected SNC rate also used the initially unlinked deaths in the numerator. Swiss reference rates were calculated with all death certificates (numerator) and the midyear reference population of Swiss Federal Statistical Office (denominator).

Figure 1 shows gender-specific all cause, all cancer, all cardiovascular, and suicide mortality rates 1991–2007 for the reference and SNC including and excluding unlinked deaths for age group 85+. For all cause mortality the uncorrected SNC rates underestimated the reference rates by 9.6% on average (range 2.4% - 17.9%). For all cancer the relative difference was similar (mean relative difference of 9.2% (range 2.4% - 18.1%), with less pronounced underestimation in years nearer the censuses and in the years 2001 to 2007 (range 2.4% - 12.5% compared to 5.5% - 17.9% in the years 1991 to 2000). Underestimation in the age-group 75–84 (see Additional file 1: Figure S1) was less pronounced than in the 85+ age-group: on average by 5.3% (range 2.4% - 7.4%) for all cause and by 4.6% (range 2.0% - 7.2%) for all cancer mortality. The SNC rates calculated after allocation of unlinked deaths were nearly identical to the reference rates. The mean and range of the relative difference over all years in the age-group 85+ was 0.3% (−1.4% to +1.8%) for all cause, 0.3% (−1.3% to 1.5%) for all cancer mortality.

### Accounting for change in official cause of death coding policy

**Figure 2:**
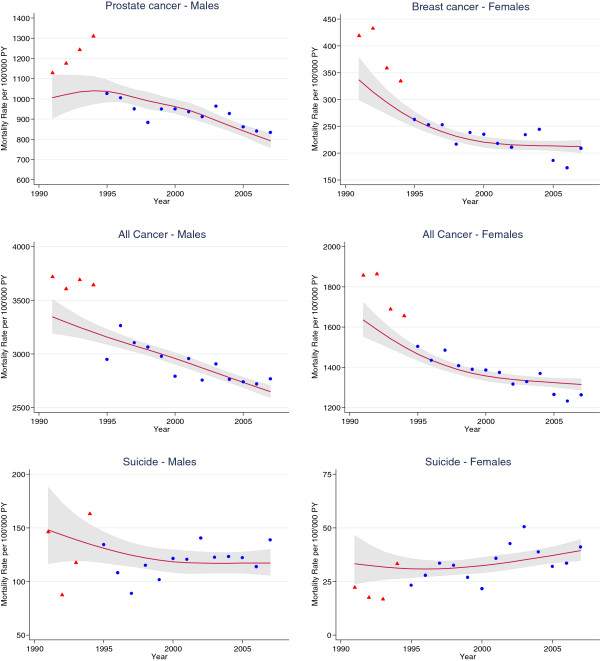
**Time trends of mortality rates in the Swiss population aged 85 and older for selected causes of death**, **accounting for the change in official cause of death coding policy.** Red triangles: observed rate in years 1991–1994, blue dots: observed rates in years 1995–2007, red line: modeled rate.

Figure 2 shows calendar trends in SNC mortality rates including the initially unlinked deaths for prostate, breast and all cancers as well as suicide for males and females in the 85+ age-group (Additional file 2: Figure S2 shows the trends for the 75–84 age-group). The rates for prostate and breast cancer dropped substantially between 1994 and 1995 with the change in cause of death coding policy. The factor by which the prostate cancer mortality rates in the years 1990 to 1994 have to be multiplied to be comparable to the rates in 1995 and later was 0.84 (95% CI: 0.77 - 0.93) in the 85+ age group, i.e. a 16% (95% CI: 7% - 33%) reduction in rate due to the change in coding policy (Table 3). For prostate cancer the multiplication factor in the age group 75–84 years was similar to the one in the 85+ age group. For breast cancer, the multiplication factors for age groups 75–84 (0.99; 95% CI: 0.90 - 1.09) and 85+ years (0.79; 95% CI: 0.72 -0.88) were distinctly different. For suicide in males almost no impact of official cause of death coding policy change was observed.

**Table 3:** Multiplication factors* **and 95**% **confidence intervals for selected causes of death comparing calendar years after 1995**–**2007 with years 1991**–**1994 in Switzerland**^Ϯ^

**Cause of death**	**Age group**	
	**75-84**	**85+**
**Females**		
All cancer	0.971 (0.931 - 1.012)	0.888 (0.850 - 0.929)
Breast	0.987 (0.896 - 1.087)	0.792 (0.716 - 0.877)
Suicide	0.975 (0.745 - 1.277)	1.422 (1.025 - 1.972)
**Males**		
All cancer	0.891 (0.859 - 0.925)	0.893 (0.854 - 0.933)
Prostate	0.798 (0.731 - 0.871)	0.843 (0.767 - 0.926)
Suicide	1.026 (0.849 - 1.240)	1.093 (0.869 - 1.375)

* Derived from Poisson regression.

^Ϯ^ Reading example: The multiplication factor of 0.792 for breast cancer in the 85+ old women means that the rate calculated in the year 1994 would need to be multiplied by 0.792 to be comparable to rate calculated in 1995.

### Comparison of relative mortality

In Table 4 we present results from multivariable Cox regression models for the gender-specific association of education with all cause, all cancer, all cardiovascular, and suicide mortality in the 85+ age group. Results for educational level hardly differed between analyses including or excluding unlinked deaths. Similarly, Cox regressions for the association of nationality (Additional file 3: Table S1) or marital status (Additional file 4: Table S2) with all cause, all cancer, all cardiovascular, and suicide mortality showed essentially identical hazard ratios when rounded to one digit after the decimal point.

**Table 4:** **Hazard ratios and 95**% **confidence intervals** (**CI**) **for mortality by gender and education** (**with**/**without unlinked deaths**) **in age group 85 years and older**

**Cause of death**	**Gender**	**Educational attainment**	**HR(95% CI)excluding unlinked SNC deaths**	**HR(95% CI)including unlinked SNC deaths**
All cause	Females	Compulsory or less	1.05 (1.04 - 1.06)	1.06 (1.05 - 1.07)
		Secondary education	1	1
		Tertiary education	0.91 (0.89 - 0.94)	0.92 (0.89 - 0.94)
	Males	Compulsory or less	1.02 (1.01 - 1.04)	1.04 (1.02 - 1.05)
		Secondary education	1	1
		Tertiary education	0.91 (0.89 - 0.92)	0.90 (0.88 - 0.92)
All cancer	Females	Compulsory or less	1.01 (0.98 - 1.04)	1.02 (0.99 - 1.05)
		Secondary education	1	1
		Tertiary education	0.95 (0.87 - 1.04)	0.95 (0.88 - 1.04)
	Males	Compulsory or less	0.96 (0.93 - 0.99)	0.98 (0.95 - 1.01)
		Secondary education	1	1
		Tertiary education	0.94 (0.90 - 0.98)	0.94 (0.90 - 0.98)
All cardiovascular	Females	Compulsory or less	1.06 (1.04 - 1.07)	1.07 (1.05 - 1.08)
		Secondary education	1	1
		Tertiary education	0.90 (0.86 - 0.93)	0.90 (0.87 - 0.94)
	Males	Compulsory or less	1.03 (1.01 - 1.05)	1.04 (1.03 - 1.06)
		Secondary education	1	1
		Tertiary education	0.91 (0.88 - 0.93)	0.90 (0.88 - 0.92)
Suicide	Females	Compulsory or less	0.65 (0.54 - 0.78)	0.67 (0.56 - 0.81)
		Secondary education	1	1
		Tertiary education	1.89 (1.35 - 2.64)	1.84 (1.32 - 2.56)
	Males	Compulsory or less	0.95 (0.81 - 1.11)	0.95 (0.81 - 1.11)
		Secondary education	1	1
		Tertiary education	1.12 (0.92 - 1.36)	1.13 (0.94 - 1.37)

HR, hazard ratio.

Multivariable Cox proportional hazard models controlled for nationality, marital status, mother tongue, religion, urbanization (place of residence), calendar year, ICD coding.

## Discussion

Mortality rates calculated in the SNC, a large population-based study with mortality follow-up ascertained through probabilistic record linkage, showed substantial differences when compared to official mortality statistics from the Swiss Federal Statistical Office (SFSO) as illustrated for all cause, all cancer, all cardiovascular, and suicide mortality. The discrepancies were removed after including the initially unlinked deaths through pragmatic linkage that only required matching for gender, age in years and geographical region but not community of residence. The lower levels of agreement of information on census and on death certificate for key variables showed that this method of allocating unlinked deaths resulted in much less reliable links than the initial more refined SNC linkage.

Changes in official cause of death coding policies must be accounted for when describing time trends of cause-specific absolute mortality rates. We achieved this by incorporating a specific parameter for the change in official cause of death coding policy in Poisson regression models with flexible restricted cubic splines to model time trends [20,21]. This allowed us to quantify the impact of the change in Switzerland and to estimate a multiplication factor by which cause-specific mortality rates in the years preceding 1995 would need to be multiplied to be comparable to those from 1995 onwards while flexibly accounting for existing time trends. Our approach integrally quantifies a sudden change in cause-specific mortality from 1994 to 1995. With our method it is not possible to disentangle the effect of the change in ICD coding form other possible causes for mortality changes occurring at the same time. Still, the interpretation of this multiplication factor is similar to the comparability ratio which has been estimated in bridging studies in the US and UK for the change of cause of death coding from ICD-9 to ICD-10 [10-13]. The comparability factor was estimated in two steps, first coding the same death certificates by both coding systems and then by dividing the number of deaths due to a certain cause (e.g. prostate cancer) as classified by ICD-10 by the number of deaths due to this cause as classified by ICD-9 [10-13]. Similar to our multiplication factor, the comparability ratio may be used to adjust cause-specific mortality rates classified by the earlier coding system for comparison with cause-specific mortality rates classified under the later coding system [10]. In the US and the UK comparability ratios clearly different from 1 were observed for deaths due to pneumonia with values of 0.70 for the US and 0.62 for England and Wales [10,12]. In contrast to the Swiss situation with multiplication factors of less than 0.9 for breast and prostate cancer in the 85+ age group, comparability ratios for breast (1.01 in US, 1.03 in England and Wales) and prostate cancer (1.01 in US, 1.04 in England and Wales) were close to 1 in the US and in England and Wales, with hardly any variation across age groups [10,11]. Variation of the comparability factor across age groups was however observed for deaths due to ischemic heart disease and myocardial infarction in England and Wales, with 0.946 for deaths in women under 75 years of age and 0.894 for women aged 85 years and older [13]. In Switzerland, no such bridging studies were conducted.

We examined hazard ratios to gain an understanding of the potential impact on results when including the pragmatically linked deaths in analyses of the SNC. We considered various outcomes (all cause, all cancer, all cardiovascular, and suicide mortality) and several independent variables (education, marital status, and nationality). These analyses reflected common mortality outcomes and important socio-demographic determinants of mortality. In all these analyses hazard ratios were very similar when including or excluding the unlinked deaths, regardless of the chosen outcome. As Greenland et al. explain [22], in some situations measurement error in the form of non-differential misclassification of a binary outcome variable (e.g. death yes/no) does not result in biased risk ratios. This happens when specificity of outcome assessment is 100% and sensitivity is the same across exposure levels. Including deaths linked to census records with perfect agreement on several identifying variables will result in a high specificity (close to 100%) of outcome ascertainment, but errors in identifying information such as marital status or community of residence will result in a sensitivity below 100%.

In the SNC, the proportion of initially unlinked deaths varied somewhat by educational attainment, marital status and nationality. Sensitivity of outcome ascertainment was thus not the same across exposure levels and one would expect that hazard ratios for these exposures might be biased [23]. By including the pragmatically linked deaths we improve sensitivity but also reduce specificity of outcome ascertainment, which also will bias results from survival analyses if sensitivity and specificity vary by levels of exposure. The way we included the initially unlinked deaths guarantees that the links are correct with regard to age (within 1 year) and sex and region of residence within Switzerland, and no bias is therefore to be expected for these exposures. In the initial and the additional pragmatic linkage we could not match on education, a powerful predictor of mortality [24-26] because education is not recorded on the death certificates. Therefore we cannot know whether sensitivity and specificity of mortality ascertainment in the SNC varied by educational level. However, the very similar results when including or excluding the initially unlinked deaths in the models for education can be interpreted in two ways. First that the level of unlinked deaths was so low that results could hardly been affected when including them, or second that the unlinked deaths did not importantly change sensitivity and specificity of mortality ascertainment by educational level.

Our study has several strengths and limitations. The main strength is that the rates and models were based on one of the largest longitudinal datasets worldwide [15] and included a long follow-up period (17 years). Several limitations result from the SNC’s reliance on routine mortality data for outcomes. First, the official underlying cause of death might not be 100% accurate. This limitation is common to all studies that rely on cause of death information provided by a national death registry. The underlying cause of death describes the “disease or injury which initiated the train of morbid events leading directly to death”, or “the circumstances of the accident or violence which produced the fatal injury” [17] and its determination may be difficult for deaths in which a number of diseases and conditions are involved. A further limitation might be that mortality rates for immigrants and foreigners may be under or over estimated because of informative censoring. This could happen if older individuals tend to return to their countries of origin after retirement and if returning to the country of origin is prognostic for death. This bias would also affect the official mortality rates for persons of foreign nationality reported by the SFSO. The extent of this potential bias cannot be assessed because mortality follow-up of persons moving out of Switzerland is not possible.

## Conclusion

In conclusion, unlinked death records and changes in official cause of death coding policy pose methodological challenges in large population-based linkage studies with follow-up over decades. We showed that correction for both unlinked deaths and changes in coding policy over time is required for an accurate description time trends of absolute mortality rates. We presented a two step approach for performing this correction by first pragmatically linking the unlinked deaths and then analyzing time trends with flexible regression models. We also showed that, in the SNC, relative mortality estimates (i.e. hazard ratios) were not affected by including the unlinked deaths. We recommend that linkage studies routinely conduct sensitivity analyses comparing results including and excluding unlinked deaths. It would be helpful to see how this method performs in other population-based linkage studies.

## Competing interest

The authors declare that they have no conflict of interest. The corresponding author had full access to all the data in the study and had final responsibility for the decision to submit for publication.

## Authors’ contribution

MZ conceived the additional linkage of unlinked deaths and the use of splines with ICD coding parameter in the Poisson regression model for the analysis of time trends, and finalized the manuscript. KS did the additional linkage, the data analysis and the writing of the first draft. CK, AS and ME contributed to the writing of the manuscript. All authors contributed to the interpretation of the results and approved the final version of the manuscript.

## Pre-publication history

The pre-publication history for this paper can be accessed here:

http://www.biomedcentral.com/1472-6947/13/1/prepub

## Supplementary Material

Additional file 1 **Figure S1.** Uncorrected and corrected SNC mortality rates for selected causes of death in comparison to the Swiss reference rates for the population aged 75 to 84 years*. Light gray triangles: uncorrected rate SNC, dark gray squares: rate SNC after allocation, black dots: reference rate. * Uncorrected SNC rates were calculated with SNC death certificates linked to census 1990 and 2000 (numerator) and exact person-time at risk (denominator). The corrected SNC rate also used the initially unlinked deaths in the numerator. Swiss reference rates were calculated with all death certificates (numerator) and the midyear reference population of Swiss Federal Statistical Office (denominator). Click here for file

Additional file 2 **Figure S2.** Time trends of mortality rates in the Swiss population aged 75–84 years for selected causes of death, accounting for the change in official cause of death coding policy. Red triangles: observed rate in years 1991–1994, blue dots: observed rates in years 1995–2007, red line: modeled rate. Click here for file

Additional file 3 **Table S1.** Hazard ratios and 95% confidence intervals (CI) for mortality by gender and nationality (with/without unlinked deaths) in age group 85 years and older. Multivariable Cox proportional hazard models. Controlled for education, marital status, mother tongue, religion, urbanization (place of residence), calendar year, ICD coding. Click here for file

Additional file 4 **Table S2.** Hazard ratios and 95% confidence intervals (CI) for mortality by marital status (with/without unlinked deaths) in age group 85 years and older.x ‡ Married including couples living apart. Multivariable Cox proportional hazard models controlled for nationality, education, mother tongue, religion, urbanization (place of residence), calendar year, ICD coding. Click here for file
