# Supplementary material for: Impact of unlinked deaths and coding changes on mortality trends in the Swiss National Cohort
Source: BMC Med Inform Decis Mak. 2013 Jan 4;13:1. doi: 10.1186/1472-6947-13-1 (PMC3547805; doi:10.1186/1472-6947-13-1)
Supplement: Additional file 1 — Figure S1. Uncorrected and corrected SNC mortality rates for selected causes of death in comparison to the Swiss reference rates for the population aged 75 to 84 years*. Light gray triangles: uncorrected rate SNC, dark gray squares: rate SNC after allocation, black dots: reference rate. * Uncorrected SNC rates were calculated with SNC death certificates linked to census 1990 and 2000 (numerator) and exact person-time at risk (denominator). The corrected SNC rate also used the initially unlinked deaths in the numerator. Swiss reference rates were calculated with all death certificates (numerator) and the midyear reference population of Swiss Federal Statistical Office (denominator). [file 1472-6947-13-1-S1.doc]

**eFigure 1: Uncorrected and corrected SNC mortality rates for selected causes of death in comparison to the Swiss reference rates for the population aged 75 to 84 years***

Light gray triangles: uncorrected rate SNC, dark gray squares: rate SNC after allocation, black dots: reference rate

* Uncorrected SNC rates were calculated with SNC death certificates linked to census 1990 and 2000 (numerator) and exact person-time at risk (denominator). The corrected SNC rate also used the initially unlinked deaths in the numerator. Swiss reference rates were calculated with all death certificates (numerator) and the midyear reference population of Swiss Federal Statistical Office (denominator)
