# Supplementary material for: Impact of unlinked deaths and coding changes on mortality trends in the Swiss National Cohort
Source: BMC Med Inform Decis Mak. 2013 Jan 4;13:1. doi: 10.1186/1472-6947-13-1 (PMC3547805; doi:10.1186/1472-6947-13-1)
Supplement: Additional file 2 — Figure S2. Time trends of mortality rates in the Swiss population aged 75–84 years for selected causes of death, accounting for the change in official cause of death coding policy. Red triangles: observed rate in years 1991–1994, blue dots: observed rates in years 1995–2007, red line: modeled rate. [file 1472-6947-13-1-S2.doc]

**eFigure 2: Time trends of mortality rates in the Swiss population aged 75-84 years for selected causes of death, accounting for the change in official cause of death coding policy**

Red triangles: observed rate in years 1991-1994, blue dots: observed rates in years 1995-2007, red line: modeled rate
