# Supplementary material for: Impact of unlinked deaths and coding changes on mortality trends in the Swiss National Cohort
Source: BMC Med Inform Decis Mak. 2013 Jan 4;13:1. doi: 10.1186/1472-6947-13-1 (PMC3547805; doi:10.1186/1472-6947-13-1)
Supplement: Additional file 3 — Table S1. Hazard ratios and 95% confidence intervals (CI) for mortality by gender and nationality (with/without unlinked deaths) in age group 85 years and older. Multivariable Cox proportional hazard models. Controlled for education, marital status, mother tongue, religion, urbanization (place of residence), calendar year, ICD coding. [file 1472-6947-13-1-S3.doc]

**eTable 1: Hazard ratios and 95% confidence intervals (CI) for mortality by gender and nationality (with/without unlinked deaths) in age group 85 years and older**

| **Cause of death** | **Gender** | **Nationality** | **HR (95% CI) excluding unlinked SNC deaths** | **HR (95% CI) including unlinked SNC deaths** |
| --- | --- | --- | --- | --- |
| All cause | Females | Swiss  Non-Swiss | 1 0.89 (0.87 - 0.91) | 1  0.92 (0.90 - 0.94) |
|  | Males | Swiss  Non-Swiss | 1 0.87 (0.84 - 0.90) | 1  0.90 (0.87 - 0.93) |
| All cancer | Females | Swiss  Non-Swiss | 1 0.96 (0.89 - 1.04) | 1  0.98 (0.91 - 1.05) |
|  | Males | Swiss  Non-Swiss | 1 0.89 (0.82 - 0.96) | 1  0.92 (0.85 - 0.99) |
| All cardiovascular | Females | Swiss  Non-Swiss | 1 0.85 (0.82 - 0.88) | 1  0.87 (0.84 - 0.90) |
|  | Males | Swiss  Non-Swiss | 1 0.83 (0.78 - 0.87) | 1  0.84 (0.80 - 0.88) |
| Suicide | Females | Swiss  Non-Swiss | 1 1.07 (0.66 - 1.72) | 1  1.14 (0.72 - 1.80) |
|  | Males | Swiss  Non-Swiss | 1 1.12 (0.77 - 1.63) | 1  1.19 (0.83 - 1.71) |

Multivariable Cox proportional hazard models

Controlled for education, marital status, mother tongue, religion, urbanization (place of residence), calendar year , ICD coding
