# Supplementary material for: Impact of unlinked deaths and coding changes on mortality trends in the Swiss National Cohort
Source: BMC Med Inform Decis Mak. 2013 Jan 4;13:1. doi: 10.1186/1472-6947-13-1 (PMC3547805; doi:10.1186/1472-6947-13-1)
Supplement: Additional file 4 — Table S2. Hazard ratios and 95% confidence intervals (CI) for mortality by marital status (with/without unlinked deaths) in age group 85 years and older.x ‡ Married including couples living apart. Multivariable Cox proportional hazard models controlled for nationality, education, mother tongue, religion, urbanization (place of residence), calendar year, ICD coding. [file 1472-6947-13-1-S4.doc]

**eTable 2: Hazard ratios and 95% confidence intervals (CI) for mortality by marital status (with/without unlinked deaths) in age group 85 years and older**

| **Cause of death** | **Gender** | **Marital status** | **HR (95% CI) excluding unlinked deaths** | **HR (95% CI) including unlinked deaths** |
| --- | --- | --- | --- | --- |
| All cause | Females | Single  Married‡  Widowed  Divorced | 1.15 (1.13, 1.17)  1 1.13 (1.12, 1.15)  1.17 (1.15, 1.20) | 1.12 (1.10, 1.13)  1  1.11 (1.10, 1.13)  1.12 (1.10, 1.15) |
|  | Males | Single  Married‡  Widowed  Divorced | 1.04 (1.02, 1.07)  1 1.09 (1.08, 1.11)  1.08 (1.04, 1.12) | 1.10 (1.07, 1.12)  1  1.10 (1.09, 1.12)  1.07 (1.03, 1.12) |
| All cancer | Females | Single  Married‡  Widowed  Divorced | 1.04 (0.99, 1.09)  1 1.09 (1.05, 1.13)  1.11 (1.03, 1.19) | 1.04 (0.99, 1.09)  1  1.09 (1.05, 1.13)  1.08 (1.00, 1.15) |
|  | Males | Single  Married‡  Widowed  Divorced | 0.77 (0.72, 0.82)  1 1.01 (0.98, 1.04)  0.97 (0.88, 1.07) | 0.82 (0.77, 0.87)  1  1.02 (0.99, 1.06)  0.97 (0.88, 1.07) |
| All cardiovascular | Females | Single  Married‡  Widowed  Divorced | 1.15 (1.12, 1.17)  1 1.14 (1.12, 1.16)  1.12 (1.08, 1.15) | 1.11 (1.09, 1.14)  1  1.12 (1.10, 1.14)  1.07 (1.04, 1.11) |
|  | Males | Single  Married‡  Widowed  Divorced | 1.05 (1.02, 1.08)  1 1.11 (1.09, 1.13)  1.04 (0.98, 1.10) | 1.10 (1.07, 1.14)  1  1.12 (1.10, 1.14)  1.04 (0.98, 1.10) |
| Suicide | Females | Single  Married‡  Widowed  Divorced | 0.73 (0.51, 1.03)  1 1.12 (0.89, 1.42)  1.47 (1.01, 2.14) | 0.79 (0.56, 1.11)  1  1.17 (0.92, 1.47)  1.54 (1.06, 2.23) |
|  | Males | Single  Married‡  Widowed  Divorced | 0.77 (0.54, 1.09)  1 1.44 (1.24, 1.69)  1.83 (1.28, 2.61) | 0.77 (0.55, 1.08)  1  1.40 (1.20, 1.63)  1.77 (1.24, 2.51) |

‡ Married including couples living apart

Multivariable Cox proportional hazard models controlled for nationality, education, mother tongue, religion, urbanization (place of residence), calendar year , ICD coding
